# Cancer Informatics for Cancer Centers: Sharing Ideas on How to Build an Artificial Intelligence–Ready Informatics Ecosystem for Radiation Oncology

**DOI:** 10.1200/CCI.23.00136

**Published:** 2023-12-06

**Authors:** Danielle S. Bitterman, Michael F. Gensheimer, David Jaffray, Daniel A. Pryma, Steve B. Jiang, Olivier Morin, Jorge Barrios Ginart, Taman Upadhaya, Katherine A. Vallis, John M. Buatti, Joseph Deasy, H. Timothy Hsiao, Caroline Chung, Clifton D. Fuller, Emily Greenspan, Kristy Cloyd-Warwick, Samir Courdy, Allen Mao, Jill Barnholtz-Sloan, Umit Topaloglu, Isaac Hands, Ian Maurer, May Terry, Walter J. Curran, Quynh-Thu Le, Sorena Nadaf, Warren Kibbe

**Affiliations:** ^1^ Artificial Intelligence in Medicine (AIM) Program, Mass General Brigham, Harvard Medical School, Boston, MA; ^2^ Department of Radiation Oncology, Brigham and Women's Hospital/Dana-Farber Cancer Institute, Boston, MA; ^3^ Department of Radiation Oncology, Stanford University School of Medicine, Stanford, CA; ^4^ Department of Radiation Physics, M.D. Anderson Cancer Center, Houston, TX; ^5^ Abramson Cancer Center, Perelman School of Medicine, University of Pennsylvania, Philadelphia, PA; ^6^ Medical Artificial Intelligence and Automation Laboratory and Department of Radiation Oncology, University of Texas Southwestern Medical Center, Dallas, TX; ^7^ Department of Radiation Oncology, MEDomics Laboratory, University of California San Francisco, San Francisco, CA; ^8^ Department of Oncology, University of Oxford, Oxford, United Kingdom; ^9^ Department of Radiation Oncology, University of Iowa Carver College of Medicine, Iowa City, IA; ^10^ Department of Medical Physics, Memorial Sloan Kettering Cancer Center, New York, NY; ^11^ Department of Scientific Affairs, American Society for Radiation Oncology, Arlington, VA; ^12^ Department of Radiation Oncology, M.D. Anderson Cancer Center, Houston, TX; ^13^ Center for Biomedical Informatics and Information Technology, National Cancer Institute, Rockville, MD; ^14^ DNAnexus, Mountain View, CA; ^15^ Center for Informatics, Digital Vertical, City of Hope National Comprehensive Cancer Center, Los Angeles, CA; ^16^ Division of Cancer Epidemiology and Genetics, National Cancer Institute, Rockville, MD; ^17^ Cancer Research Informatics Shared Resource Facility, University of Kentucky Markey Cancer Center, Lexington, NY; ^18^ Kentucky Cancer Registry, Lexington, NY; ^19^ GenomOncology, Cleveland, OH; ^20^. MITRE Corporation, Bedford, MA; ^21^ GenesisCare, Fort Myers, FL; ^22^ Department of Radiation Oncology, Emory University, Atlanta, GA; ^23^ Cancer Center Informatics Society, Los Angeles, CA; ^24^ Department of Biostatistics and Bioinformatics, Duke University, Durham, NC

## Abstract

In August 2022, the Cancer Informatics for Cancer Centers brought together cancer informatics leaders for its biannual symposium, Precision Medicine Applications in Radiation Oncology, co-chaired by Quynh-Thu Le, MD (Stanford University), and Walter J. Curran, MD (GenesisCare). Over the course of 3 days, presenters discussed a range of topics relevant to radiation oncology and the cancer informatics community more broadly, including biomarker development, decision support algorithms, novel imaging tools, theranostics, and artificial intelligence (AI) for the radiotherapy workflow. Since the symposium, there has been an impressive shift in the promise and potential for integration of AI in clinical care, accelerated in large part by major advances in generative AI. AI is now poised more than ever to revolutionize cancer care. Radiation oncology is a field that uses and generates a large amount of digital data and is therefore likely to be one of the first fields to be transformed by AI. As experts in the collection, management, and analysis of these data, the informatics community will take a leading role in ensuring that radiation oncology is prepared to take full advantage of these technological advances. In this report, we provide highlights from the symposium, which took place in Santa Barbara, California, from August 29 to 31, 2022. We discuss lessons learned from the symposium for data acquisition, management, representation, and sharing, and put these themes into context to prepare radiation oncology for the successful and safe integration of AI and informatics technologies.

## INTRODUCTION

The Cancer Informatics for Cancer Centers (Ci4CC) is the premier professional society for cancer precision medicine and informatics and provides a focused forum for professionals from National Cancer Institute (NCI)–Designated and Community Cancer Centers emphasizing precision medicine, data science, health care informatics, translational research, and digital platforms. The summer 2022 Ci4CC Symposium, titled Precision Medicine Applications in Radiation Oncology, was developed as a collaboration of investigators from NRG Oncology, American Society for Radiation Oncology (ASTRO), NCI, cancer centers, and Ci4CC to focus on innovative precision medicine and informatics in the field of radiation oncology. Dr Quynh-Thu Le from Stanford University and Dr Walter J. Curran from GenesisCare chaired the symposium, which covered four themes: (1) biomarker development for precision radiation oncology; (2) decision support algorithms in radiation oncology and their impact on clinical practice and trials; (3) the role of machine learning and AI in the radiation therapy workflow; and (4) the use of new imaging tools and advanced analytics in radiotheranostics. In addition, current efforts for establishing data standards and informatics infrastructure from academic, industry, and government stakeholders at local and national levels were discussed. The goal of this meeting was to highlight opportunities and challenges in these areas, and to connect academic and industry researchers who work in these fields within and outside of radiation oncology to develop new collaborations to improve the care and outcome of patients treated with radiotherapy (RT).

Table 1 highlights some of the emerging AI opportunities for radiation oncology discussed during this symposium, along with unmet informatics needs for their promise to be realized.

**TABLE 1.**
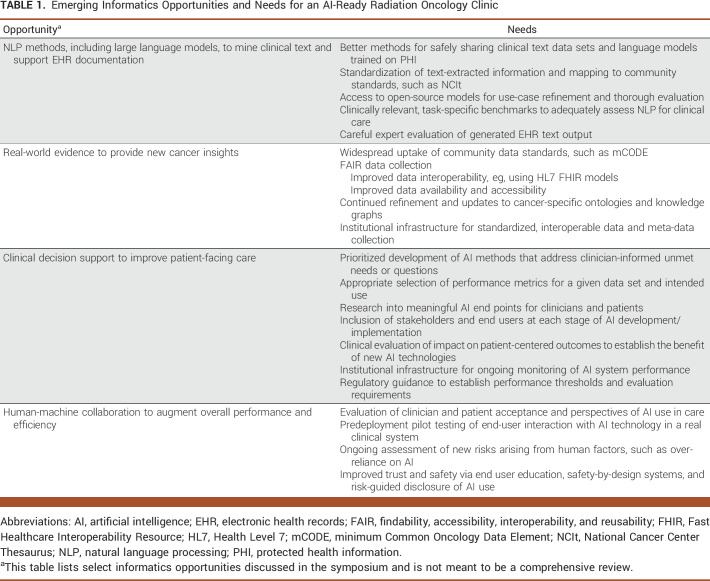
Emerging Informatics Opportunities and Needs for an AI-Ready Radiation Oncology Clinic

## BIOMARKER DEVELOPMENT

Recent years have seen the development of multiple biomarkers to guide precision radiation oncology, developed from the many clinical and biological data types collected on patients during their care. Dr Felix Feng from University of California San Francisco discussed how AI-based analysis of pathologic images can improve upon traditional pathology review to guide treatment recommendations that are personalized to each patient's disease. Dr Max Diehn from Stanford University presented on genomic-based biomarkers of RT resistance, including tumor mutations that can predict recurrence after RT^1,2^ and circulating tumor DNA dynamics that can measure minimal residual disease after definitive therapy^3^ in lung cancer. Dr Joanne Weidhaas of University of California Los Angeles discussed microRNA-based biomarkers for RT patient selection. MicroRNA mutations can predict cancer treatment toxicity^4^ as well as RT response.^5^ These biomarkers from diverse data types may improve the overall risk-to-benefit ratio of cancer treatment.

Dr Joseph Deasy from Memorial Sloan Kettering Cancer Center described how the search for quantitative biomarkers is often confounded by the large-scale nature of the underlying genomics. Network data representations, where each node of the network represents a different feature of the system, combined with Wasserstein/earth-mover distance measurements between samples, have emerged as a powerful tool to characterize cancer variability.^6^ These methods seek to incorporate information sharing between network components without detailed molecular modeling. In particular, these methods can be used to model large-scale genomics (mRNA, copy-number variation, and methylomics),^7,8^ as well as radiomics,^9^ and can be combined with other approaches to better understand subtypes,^10,11^ prognosis,^12^ and treatment response.^13^

## DECISION SUPPORT ALGORITHMS

The ASTRO recognizes the potential of advancing computation and AI for precision radiation oncology, as highlighted in a joint collaborative panel between the ASTRO, the NCI, and the Department of Energy (DOE). Speakers included Dr Caroline Chung and Dr Clifton (Dave) Fuller, both from the MD Anderson Cancer Center, Dr Christine Chalk from the DOE, Dr Emily Greenspan from the NCI, and Dr H. Timothy Hsiao (Moderator) from the ASTRO. ASTRO officially included Big Data Analytics and Artificial Intelligence as a strategic area in the ASTRO Research Agenda starting in 2020.^14^ The ASTRO Science Council has also been collaborating with the NCI and DOE and produced a collaborative workshop in 2021 and a publication Predictive Radiation Oncology—A New NCI-DOE Scientific Space and Community in 2022.^15^ To achieve the goals of precision medicine and learning health systems, ASTRO places emphasis on collecting, processing, and curating patient-level radiation oncology data. In addition, ASTRO recognizes the potential of AI to help realize the promise of precision medicine and modernize healthcare in three major areas: (1) disease prevention, (2) personalized diagnosis, and (3) personalized treatment. AI tools must remain a priority in radiation oncology research and for future integration into clinical workflows.

In addition to the high-level strategic vision of ASTRO for advancing AI, examples of ongoing efforts developing decision support algorithms for radiation oncology and how they may affect practices along the cancer care and research continuum were provided throughout the symposium and serve as early examples of the many ways AI may transform RT. Dr Michael Gensheimer from Stanford University discussed machine learning to improve advance care planning (ACP) conversations for patients with cancer. Machine learning is increasingly being used to optimize care delivery for patients with cancer. One area of recent focus is ACP.^16^ Radiation oncologists frequently see patients with poor prognosis and are involved in end-of-life care, and hence have the opportunity to improve rates of ACP in radiation oncology. Studies have explored training nonphysicians, such as lay health workers, care coaches, and nurse navigators, to assist with ACP, but it can be hard for these staff to identify which patients to approach for conversations. At Stanford, an automated survival prediction model was developed to help providers identify high-risk patients and prioritize care. This model was trained on data from approximately 15,000 Stanford patients with metastatic cancer and outperformed both physicians and traditional models in predicting 1-year survival.^17^ A pilot quality improvement study implemented care coaches and weekly automated emails showed a sustained increase in ACP and prognosis documentation, as well as high provider engagement.^18^ To learn about physician and patient perspectives, Dr Gensheimer's team conducted a qualitative study of physicians and patients in which they were shown a model report for a real anonymized patient.^19^ Physician interviews revealed that a lack of patient-specific data hinders prognosis conversations and that concerns over validation and explainability are barriers to use. Patient interviews indicated that prognosis conversations can be empowering, and patients trust computer models that use a wide range of predictors. However, fear of giving up or denial of a finite lifespan can hinder these conversations.

Dr Danielle Bitterman from Brigham and Women's Hospital/Dana-Farber Cancer Institute spoke about how natural language processing methods may improve outcome assessment in radiation oncology. Outcomes such as cancer-specific survival, patterns of disease failure, and short- and long-term adverse events are critical for clinical trials and health outcomes research and remain very difficult and resource-intensive to collect reliably over long periods of time. Although there has been widespread uptake of electronic health records (EHRs), our ability to generate real-world evidence has thus far been limited, in part because many cancer outcomes are documented only in clinical text and cannot be automatically abstracted for downstream analysis. Recent advances in natural language processing, especially neural-based pretrained language models, offer an avenue to automate this process.^20,21^ Dr Bitterman presented emerging efforts to automatically extract cancer outcomes using these AI methods.^22,23^ Natural language processing has also been shown to extract acute RT toxicities^24,25^ and may provide new data on nonmedical outcomes after RT.^26^

In addition, Dr Olivier Morin from the University of California San Francisco discussed the potential and challenges of predictive modeling using real-world data from EHRs. EHR data suffer from:

1. Poor standardization: hospitals store their data differently.
2. Siloed structured and unstructured data: medical information in an EHR, radiation information in an Oncology Information System, images in a picture archiving and communication system, and molecular testing in its own system.
3. Incomplete patient treatment outcomes: no improvement can be made if important data are not recorded.

Additionally, EHRs presently have no feedback mechanisms to assess/ensure data quality, learn from past experience, and update for real-time changes in cancer management. Better utilization of digital health data will enable us to expand the learning pool while simultaneously reducing confounding factors such as selection bias. With the emergence of statistical learning and large language models, AI tasks in oncology such as workflow optimization, feature extraction, detection of clinical trial eligibility, and risk stratification can be developed to provide decision support using historical data. Dr Morin's group is developing a framework and methods to understand and predict the dynamic changes in surviving groups. Their framework currently consists of thousands of individuals with cancer and millions of data points over a decade of data recording. They reported a proof-of-concept analysis using this infrastructure, which identified the Framingham risk score to be robustly associated with mortality among individuals with early-stage and advanced-stage cancer, a potentially actionable finding from a real-world cohort of individuals with cancer. Finally, they showed how natural language processing of medical notes could be used to continuously update estimates of prognosis as a given individual's disease course unfolds.

## AI FOR THE RADIOTHERAPY WORKFLOW

An overview of practical applications of AI in radiation oncology, especially AI-based segmentation tools, was provided by Dr John Buatti from the University of Iowa. One known weakness of manual tumor and normal structure contouring is creating consistent contours, even among human experts. Inconsistency among physicians and even for the same physician contouring structures on identical images at different times creates errors and limits the ability to consider true tumor control probabilities or normal tissue complication probabilities.^27^ In addition, this inconsistency limits our ability to interpret RT clinical trials, especially when central plan review is lacking.^28^ Furthermore, contouring is extremely time-consuming and hence expensive. AI has been shown to efficiently and consistently identify both tumors^29^ and normal structures^30-34^ using a variety of different algorithms and approaches, offering a potential mitigation to the challenges of manual contouring. Daily workflow application in radiation oncology remains at extremely early phases but promise to revolutionize our approach to planning. AI-based segmentation is currently facilitating the deployment of real-time adaptive RT, illustrated in magnetic resonance imaging (MRI)-Linac systems that obtain daily MR images to create a daily plan optimized for the location and size of the tumor and surrounding critical structures. Full deployment of these AI-based technologies is only just beginning but holds promise to improve RT quality in the long term.

Other emerging workflow applications were discussed throughout the symposium. For example, AI-based technologies could enable image reconstruction to improve tumor delineation and response assessment over time,^35^ automate RT planning from computed tomography and MRI simulation scans,^36,37^ and improve image-guided RT by facilitating tracking from onboard imaging.^38^ Deep learning–based analyses of imaging over time may also support monitoring strategies for more quantitative assessments of tumor progression.^39^

Importantly, while the past years have seen a proliferation of research into methods to automate the RT workflow, clinical translation and research into its clinical effectiveness has lagged.^27^ Dr Steve Jiang of University of Texas Southwestern Medical Center discussed how variations in physician practice pose a crucial challenge to AI implementation. These variations can be classified into two types—type A and type B. Type A variations stem from a lack of experience or information, often representing data noise that, if reduced, can facilitate standardization of care. One of the ways AI can address this issue is through decision support tools, presenting relevant information and suggesting optimal treatment options. However, type B variations, which are variations arising from the personalization and art of clinical medicine, need to be acknowledged and incorporated when developing and deploying AI tools. AI tools should be flexible and adjustable, giving physicians the space to use their clinical judgment and expertise. It is through this recognition of type B variations that trust can be fostered between physicians and AI tools, encouraging a collaborative relationship that promotes shared decision making. Creating AI tools that do not attempt to replace human expertise but work alongside it will ensure patient-centered care that respects the individuality of physician practices.

Additional challenges to achieving the promises of AI for the RT workflow were discussed by Dr Ceferino Obcemea from the NCI, including the acceptability of performance failures in critical safety domains, data leakage and reproducibility,^40^ and a need for more explainable methods and uncertainty quantification. In addition, individual performance metrics such as area under the curve^41^ and accuracy^42^ are not sufficient to comprehensively evaluate a clinical AI application. The best statistical analysis depends on the data, its distribution, and model end use. Multiple complementary statistical analyses are needed to understand model uncertainty and performance.^43^ Multidisciplinary collaboration between clinicians, computer scientists, experts in human-computer interaction, and statisticians will be needed for the effective, safe, and trustworthy integration of AI into RT workflows.

## RADIOTHERANOSTICS

Radiotheranostics, the use of a cancer-seeking ligand that can incorporate radionuclides suitable for imaging or therapy, is an expanding branch of cancer medicine. Because therapeutic radiopharmaceuticals (and/or their companion diagnostics) have imageable radioactive emissions, it is possible to track uptake and clearance kinetics in tumor sites and organs at risk in individual patients. Although personalized dosimetry, with prescribed radiation dose deposition in tumor planned within normal organ constraints, is the norm with brachytherapy and external-beam RT, Dr Daniel Pryma from the University of Pennsylvania explained that it is unclear whether that approach is optimal with therapeutic radiopharmaceuticals. Accurate dosimetry as well as a dearth of data to support a benefit of personalized dose selection remains a challenge. The benefits of population-based dose selection were reviewed.

Conversely, Dr Katherine Vallis from Oxford University discussed that reports indicate a clear link between tumor radiation absorbed dose and likelihood of response, strengthening the drive toward individualized dosing.^44^ Personalized dosimetry, however, requires serial scan acquisitions to allow tumor and normal organ cumulative dose calculations, a process that is resource-intensive and costly. Recent AI innovations have the potential to accelerate the dosimetry workflow, making it less onerous and reducing the need for user intervention.^45^ Current efforts are aimed at developing models that use pretreatment images to predict therapy dosimetry, which would eliminate the need for multiple post-treatment imaging sessions. Because the number of patients currently treated with this radiotheranostics is relatively small, intercenter cooperation and harmonization of clinical protocols will be necessary to enable sufficiently large data sets for model training. Finally, a call to arms was issued to the informatics community to help reduce the technical burdens of dose personalization and improve the accuracy and reproducibility with the hope that in the future, trials could rigorously test the effects of dose personalization on patient outcomes.

## DATA STANDARDS

Data standards for oncology are key to achieving the full promise of AI and were a running theme throughout the symposium. Interoperability and creating representative data sets present considerable challenges that must be addressed before AI can be implemented into routine cancer care. May Terry from the MITRE Corporation led a workshop on the minimum Common Data Elements (mCODE),^61^ which is a consensus-driven standard on the basis of the Health Level 7 (HL7) Fast Healthcare Interoperability Resources (FHIR) specification.^46^ mCODE comprises a core set of data elements needed to care for every patient with cancer. Through the HL7 CodeX FHIR Accelerator, a member-driven and collaborative community, mCODE is tested and piloted in real-world use cases such as clinical trial matching, radiation oncology, previous authorization, cancer registry reporting, and genomics data exchange. This community-driven standardization of oncology data facilitates the capture and exchange of high-quality and interoperable oncology data that are essential for deriving insights and improving clinical outcomes.

The ASTRO Clinical Affairs and Quality Council has been actively supporting cross-sector initiatives such as mCODE and CodeX.^47^ CodeX use cases are currently basic, day-to-day focused, but the standards that result from these initiatives will lay critical foundations for an AI-ready informatics radiation oncology ecosystem.

Of note, since the symposium, the American Association of Physicists in Medicine developed the Operation Ontology for Oncology (O3), which is a standardized ontology for clinical data, social determinants of health, and other RT concepts and relations.^48^ O3 extends and is interoperable with existing data standards, and was developed to address quality, safety, accreditation, billing, and research needs in radiation oncology. Taken together, these efforts will facilitate the collection and sharing of findability, accessibility, interoperability, and reusability (FAIR) data to enable research.

Dr Jill Barnholtz-Sloan and Dr Umit Topaloglu of the NCI discussed national efforts in data standards. To minimize bias and improve fairness at the data level, NCI is focusing on data availability, quality, and conformance as well as multisite data harmonization by investing heavily on semantic infrastructure. Existing ontologies and terminologies define cancer-related concepts and their relationships, which help with reasoning and semantic query. The NCI semantics team is currently focused on establishing a graph representation of the cancer-specific ontologies to form knowledge graphs that can be consumed by many AI methods and other informatics approaches. Similarly, more than 70,000 Common Data Elements (CDEs) with Clinical Data Interchange Standards Consortium^49^ terminology annotations are being used by NCI-sponsored studies and clinical trials. CDEs improve data quality and completeness as well as enable data interoperability. Additionally, in coordination with National Center for Advancing Translational Sciences, US Food and Drug Administration (FDA), and Office of the National Coordinator (ONC), NCI facilitates common data model mapping and harmonization efforts that use FHIR and Biomedical Research Integrated Domain Group^50,51^ models for harmonization and registers models with the cancer Data Standards Repository as a service. Such services enable interoperability across four common data models and provide a single querying platform for cancer research.

## INFORMATICS INFRASTRUCTURES

A learning health system will require enhanced and new data science infrastructures at the local and national levels. For example, Drs David Jaffray and Caroline Chung from M.C. Anderson Cancer Center discussed challenges to successful implementation of AI methods, including nongeneralizability of models outside of populations they were trained on, poor reproducibility because of a lack of standardized data acquisition, and performance drift where models degrade over time. Because data collection and health care practices are constantly evolving in real-world clinical environments, there is a need to monitor algorithms in an ongoing fashion. In addition, the community would benefit from demonstrating the impact of AI methods on survival and other patient-centered outcomes. New organizational frameworks for coordinated and standardized data and meta-data collection, as well as lifecycle algorithm management that adhere to regulatory guidelines, will hasten the clinical translation of safe, effective, and impactful AI decision support algorithms.

Drs Barnholtz-Sloan and Umit Topaloglu presented on infrastructure efforts at the national level. With the Cancer Moonshot Blue Ribbon Panel,^52^ Health and Human Services (HHS) Priorities,^53^ and the new National Cancer Plan^54^ all recommending that a National Cancer Data Ecosystem be built and sustained, the Center for Biomedical Informatics and Information Technology at the NCI has built a foundation for this Ecosystem with the Cancer Research Data Commons (CRDC).^55^ The vision for the National Cancer Data Ecosystem includes increased collection and use of data by implementing plans for access, search, and retrieval of multimodal data sets to break down silos and expand data sharing for maximal impact on cancer care and prevention. The CRDC provides the cancer research community with state-of-the-art analysis and interoperability tools in a flexible cloud-based computational environment. The CRDC currently offers genomic, proteomic, imaging, and canine/human comparison data as well as many specialized data sets via the Cancer Data Service.^56^ Coming soon to the CRDC will be data from NCI-sponsored clinical trials and population science study data. In addition, the NCI is working on plans for a new Cancer Data Science Hub, which will allow for ease of data submission, will increase the variety of data within the CRDC, and create intuitive, easy-to-use search and analysis tools.

Next, informatics leaders presented on a range of informatics and data shared resources at NCI Cancer Center Support Grant (CCSG) Comprehensive Cancer Centers. Isaac Hands from the University of Kentucky, Markey Cancer Center, described how the Markey Cancer Research Informatics Shared Resource Facility (CRI) is building a cancer research data commons to address the cancer burden in central Appalachia on the basis of a Local Data Commons architecture.^57^ The central region of Appalachia, made up primarily of counties in Kentucky and West Virginia, has some of the highest cancer incidence and mortality in the United States.^58^ Some studies have suggested differences in the genetics of patients with cancer in Appalachia versus the rest of the United States,^59^ but have been limited to relatively small samples of patients and require an analysis of larger populations in Appalachia. The Markey Cancer Center informatics effort hopes to help answer questions about why the Appalachian population bears a higher burden of cancer than other parts of the United States—the first focused on Appalachian patients with cancer. The central Appalachian data commons currently hosts population-based registry data on more than 739,000 patients with cancer from the SEER Kentucky Cancer Registry, pathology reports from more than 50 regional laboratories, and more than 7,000 genomic sequencing results linked to the patients in the registry. The central Appalachian data commons uses a custom instance of the cBioportal^60^ genomics discovery platform to provide a user-friendly cohort discovery and data exploration tool for researchers to identify cohorts of interest. Once a cohort has been identified, CRI will review requests for enhanced data sets as part of a data governance process that protects patient privacy while allowing access to critical raw data elements. Researchers can find more information on the CRI website.^62^ Other topics discussed included clinical pathways in precision medicine and applied AI and data science at City of Hope Comprehensive Cancer Center (Dr Deron Johnson and Nasim Eftekhari from City of Hope) and Cancer Center CCSG IT support (Dr Michael Townsend from The James Comprehensive Cancer Center at Ohio State).

In addition to these efforts at health care institutions and government, industry partnerships may also support the expanding informatics needs in clinical medicine. An industry keynote from DNAnexus by Drs Kristy Cloyd-Warwick and Sam Westreich, presenting on work performed with City of Hope's Samir Courdy, described how POSEIDON, which is an enterprise-wide data and analytics platform ready to power the AI precision medicine revolution, has been established at City of Hope to support AI exploration and utilization. Built on DNAnexus with custom features and functionality created by City of Hope Research Informatics, POSEIDON unifies imaging, imaging metadata, and comprehensive germline and somatic genomic profiling for nearly 700,000 patients. POSEIDON addresses traditional real-world data challenges upstream. With DNAnexus as its foundation, POSEIDON has industry-leading security, access control, and governance features at its core; a flexible, scalable infrastructure that can speed through complex workflows; and an intuitive interface that is accessible to all user types. On top of this foundation, City of Hope has built powerful data harmonization and interoperability features that allow researchers and clinicians to extract tremendous value from the intersection of multimodal real-world data, enabling AI exploration and utilization. POSEIDON was built with radiation oncology use cases in mind, acknowledging the high volume of data associated with this highly technical field.

Ian Maurer from GenomOncology presented an industry keynote on GenomOncology's Precision Oncology Platform, which is an expert-curated, ontology- and rules-based knowledge base that can complement and expand the abilities of generative large language models. In the context of medical question answering, GenomOncology's ChatGPT plugin enables the language model to support biomarker annotation, interpretation, and clinical trial or therapy matching. The plugin also ensures the accuracy and relevance of answers by validating URLs, PubMed IDs, and monitoring new developments such as FDA Fast Track status and clinical trial recruiting statuses. For data extraction, GenomOncology's optical character recognition solution supports various formats, including scans and faxes, enabling the extraction of data from complex documents such as next-generation sequencing and pathology reports, and semantically linking the data to standard ontologies to ensure interoperability.

In conclusion, radiation oncology uses diverse data types, including but not limited to clinical EHR data, radiologic images, pathology, genomics, RT tumor and normal tissue structures, and dose maps—all of which are often dynamic over time. These data must be collected and curated thoughtfully and equitably for AI-based analyses to offer meaningful and reproducible insights into cancer care. The cancer informatics community has a wealth of expertise in managing such data and will take a leading role in ensuring the effective and safe transition into a new era of data-driven health care. As highlighted throughout this symposium, our community has created, and continues to build, shared resources and data standards toward this goal. Institutions should adopt these as soon as possible so that clinical data can be reliably used for advanced AI analysis, and informaticians should partner with clinical researchers, cancer clinicians, and patients to codevelop AI technologies that usher in a new era in cancer care.
